# Effectiveness of metaverse-based family sculpture and meditation in reducing psychological distress

**DOI:** 10.3389/fpsyg.2025.1649129

**Published:** 2025-09-03

**Authors:** Sang Hyun Yoo, Eun Jin Choi, Hong Sik Yang

**Affiliations:** EZNwellness Corporate Research Institute, Seoul, Republic of Korea

**Keywords:** meta-counseling, anxiety, therapeutic intervention, psychological distress, digital psychotherapy

## Abstract

**Introduction:**

The metaverse has demonstrated notable effectiveness in psychotherapeutic treatments. This study investigates the efficacy of a metaverse-based family sculpture technique in reducing psychological stress, anxiety, and depression.

**Methods:**

Traditional family sculpture methods, known for their ability to identify and address familial relationship dynamics, were adapted to a metaverse environment, facilitating immersive, nonphysical therapeutic interactions. Participants (*n* = 79) were divided into family sculpture, meditation, combined meditation and family sculpture, no-intervention, and placebo groups engaging in a wish lantern activity. Standardized tools (Generalized Anxiety Disorder-7, Patient Health Questionnaire-9, Perceived Stress Scale) measured psychological outcomes, followed by pre- and post-intervention assessments.

**Results:**

The combined meditation and family sculpture group exhibited the most significant reductions across all measures, demonstrating a synergistic effect between the two techniques. The family sculpture group effectively reduced anxiety, whereas the meditation group significantly decreased stress and depression. Satisfaction levels were the highest in the combined meditation and family sculpture group, reflecting participants’ positive perceptions of emotional stabilization and the usability of metaverse technology.

**Discussion:**

Metaverse-based interventions can expand access to psychotherapy, offering a novel and engaging platform for therapeutic innovation. Future research should explore larger sample sizes, extended intervention durations, and technical refinements to validate the long-term sustainability and effectiveness of these digital psychotherapeutic approaches.

## 1 Introduction

Psychotherapeutic techniques that delve into the interactions and dynamics among family members help to understand individual mental health. The family sculpture technique is especially salient for its effectiveness in visualizing family relationships, offering an intuitive approach to uncovering internal family dynamics (Jefferson, 1978). While traditionally practiced in offline settings, this method has recently broadened its scope of application through digital platforms such as virtual reality (VR) and the metaverse.

The metaverse, implemented as a three-dimensional immersive virtual environment, enables diverse interactions beyond physical limitations (Caponnetto et al., 2021). In psychotherapy and counseling contexts, it provides anonymity, convenience, and flexibility in visual expression, proving particularly effective in reducing emotional distress and encouraging genuine self-expression (Riva et al., 2021; Cho et al., 2024).

In psychotherapy, the metaverse has demonstrated notable effectiveness in the treatment of anxiety disorders, phobias, and post-traumatic stress disorder. VR-based exposure therapy allows patients to experience situations they fear or anxieties within a safe virtual environment, reducing psychological resistance and facilitating effective treatment (Yoo et al., 2024), as evidenced by studies on individuals with acrophobia or social anxiety disorders.

The metaverse represents progression in enhancing therapeutic accessibility. Providing an environment where therapy can be accessed without the temporal and spatial constraints of conventional settings strengthens rapport between therapists and clients (Bhardwaj et al., 2023). This innovation thus demonstrates functionality in formats such as group therapy or remote counseling and offers a new therapeutic avenue (Wiederhold and Riva, 2019).

The family sculpture technique visually represents interactions within the family of origin, offering a clear understanding of family dynamics and providing deeper insights into an individual’s inner self. This approach helps both therapists and clients to easily identify structural issues within family relationships. By facilitating the expression of emotions tied to past wounds from the family of origin, emotional distance can be reduced and closer interpersonal interactions promoted in current relationships (Kreijen and Abbing, 2024). This visualization method is especially useful in addressing communication issues between parents and children, resolving emotional detachment in couples, and overcoming broader interpersonal challenges.

The family sculpture technique is effective in facilitating the expression of suppressed emotions within family settings while enhancing understanding and strengthening familial relationships. Therapies incorporating the family sculpture technique improve emotional understanding in couples and the interaction quality in couple and family counseling sessions (von Sydow et al., 2024). Further, visualizing family structures helps clients recognize the psychological distance between family members, thus improving communication and effective problem-solving within the family (Kreijen and Abbing, 2024). This technique can also effectively address psychological challenges among adolescents by helping them to better understand their roles and positions within the family while facilitating the expression of emotional distress they experience in their relationships with parents (Brandon and Goldberg, 2017). These findings indicate that the family sculpture technique is a valuable tool in adolescent counseling, offering emotional support and fostering improvement in familial relationships.

The family sculpture technique transcends mere conversations and effectively uncovers conflicts and interactions underlying interpersonal relationships (Hamill, 2013). From a psychodynamic perspective, repressed emotions and conflicts within the family are vulnerabilities for developing anxiety disorders. This technique has therapeutic outcomes as it fosters emotional communication, resolves conflicts, and reconstructs family relationships (Guillemet and Jackson, 2019). It is proficient in revealing hidden emotional issues through nonverbal communication, thereby contributing to psychological stability and alleviating emotional challenges such as depression, anxiety, and stress.

The metaverse-based family sculpture technique has substantial potential for advancing its therapeutic utility by extending traditional offline therapy into a digital environment. In the metaverse, family members can engage in interactions that surpass physical limitations, offering therapists a unique opportunity to observe and analyze family dynamics for gaining a deeper understanding of their relationships. Onnis et al. (1994) demonstrated the effectiveness of the family sculpture technique in visualizing suppressed emotions and resolving psychological issues. However, little research has been conducted to explore how this technique can be applied in digital environments. With the growing demand for digital psychotherapeutic approaches in the aftermath of the COVID-19 pandemic, studying the effectiveness of the family sculpture technique as an insightful therapeutic tool for addressing psychological issues becomes relevant.

This study seeks to validate the effectiveness of a metaverse-based digital family sculpture technique. In the digital therapy environment, this technique becomes more intuitive and immersive, introducing innovative changes to therapy sessions dealing with emotional issues. It could provide effective treatment options for clients enduring psychological distress such as depression, anxiety, and stress. Specifically, it seeks to compare the outcomes of the metaverse-based family sculpture technique with those of metaverse-based meditation and a no-intervention control group, analyzing the differences between these approaches. Metaverse-based meditation promotes psychological stability and reduces stress, but its impact on family interactions and relationships may be limited (Bhardwaj et al., 2023). By contrast, the metaverse-based family sculpture technique directly drives changes by visually representing family relationships and fostering interaction among members. Building on these assumptions, this study suggests that the metaverse-based family sculpture technique is more therapeutic effective than both meditation and no-intervention groups.

By comparing these groups, this research aims to assess the impact of the metaverse-based family sculpture tool on a person’s mental health and evaluate its efficacy relative to meditation and no-intervention groups. These findings are expected to highlight the potential applications of metaverse technology in psychotherapy and offer valuable insights into the digital transformation of psychotherapeutic methods.

## 2 Materials and methods

Five comparison groups were established to test the effectiveness of a metaverse-based family sculpture tool in reducing workplace stress.

Standardized stress level questionnaires were used to conduct pre- and post-tests for measuring the effectiveness of each group, and qualitative interviews were conducted to evaluate family interactions and relationship changes. Statistical analysis of stress-reduction effects across the five groups validated the effectiveness of the metaverse-based family sculpture tool and quantified its differences relative to the meditation, no-intervention, and placebo groups.

### 2.1 Research procedure

#### 2.1.1 Research design

The study was designed to evaluate the stress-reduction effects of a metaverse-based family sculpture tool by establishing five comparison groups with a repeated-measures design, including pre- and post-tests. The groups comprised (1) an experimental group utilizing the metaverse-based family sculpture technique; (2) an experimental group utilizing the metaverse-based meditation technique; (3) an experimental group combining the metaverse-based meditation and family sculpture (M + FS) techniques; (4) a control group receiving no intervention; and (5) a placebo group engaging in a wish lantern activity. The therapeutic effects were analyzed through comparisons across the groups.

#### 2.1.2 Participant enrollment

Participants were recruited through public advertisements on online platforms, social media, and relevant communities. The target population included adults who were either interested in psychotherapy or required stress management. The recruitment material provided detailed information about the study’s objectives, procedures, participation process, and privacy policies. Eligibility for participation required individuals to meet two criteria: voluntary participation and access to and familiarity with the metaverse technology. The study was approved by the Public Institutional Bioethics Committee designated by the Ministry of Health and Welfare, Republic of Korea, approval no. P01-202504-01-006, dated April 7, 2025. This study collects activity data in a metaverse environment through PC-based interactions. The data collection process ensures participant anonymity and privacy by excluding personally identifiable information. This study was classified as minimal risk research, as it did not present physical, psychological, or emotional risks to participants.

A total of 80 participants were initially recruited. After excluding individuals who withdrew because of personal circumstances or lacked the required metaverse setup, 79 participants were enrolled in the study. Written informed consent, including concurrence for using personal information, was obtained from all the participants. As an expression of gratitude, each participant received a mobile coupon worth approximately 100,000 won.

#### 2.1.3 Random assignment

To enhance internal validity and ensure group comparability, participants were randomly assigned to one of the five intervention groups using a stratified randomization procedure. Stratification was based on participants’ responses to a baseline demographic questionnaire, considering key variables such as gender, age, and family composition. This method was chosen to minimize baseline group differences and evenly distribute these characteristics across groups. Randomization within each stratum was manually conducted by the research team using pre-generated lists. Although full blinding of participants was not feasible due to the nature of the interventions, outcome assessors remained blind to group assignments to reduce potential detection bias. Table 1 presents the demographic composition of each group following randomization.

**Table 1:** Participants’ demographic characteristics.

Group	Gender			Age group				
	*M*	*F*	Total	20s	30s	40s	50s	Total
No-intervention group	5	14	19	7	10	2	0	19
Wish lantern group	2	12	14	6	7	1	0	14
Meditation group	4	11	15	6	8	1	0	15
Family sculpture group	4	14	18	6	11	1	0	18
M + FS group	3	10	13	4	8	0	1	13
Total	18	61	79	29	44	5	1	79

### 2.2 Assessment tools

Standardized assessment tools were employed to evaluate stress, anxiety, and depression levels.

#### 2.2.1 Perceived Stress Scale

The Perceived Stress Scale (PSS), developed by Cohen et al. (1983), measures individuals’ perceived stress in life. It comprises ten items rated on a 5-point Likert scale (0 = almost never; 4 = very often), with total scores ranging from 0 to 40. Higher scores indicate greater levels of perceived stress. This study used the PSS to examine the impact of the metaverse-based family sculpture and meditation techniques on stress levels. The internal consistency reliability (Cronbach’s alpha) was 0.78 for the pre-test and 0.81 for the post-test.

#### 2.2.2 Generalized Anxiety Disorder-7

The Generalized Anxiety Disorder-7 (GAD-7), a seven-item scale developed by Spitzer et al. (2006), evaluates anxiety levels by measuring the frequency of anxiety symptoms that the study participants had experienced over the past 2 weeks. Each item is scored on a 4-point Likert scale (0 = not at all; 3 = nearly every day), with total scores ranging from 0 to 21. Higher scores reflect greater levels of anxiety. This study employed the GAD-7 to assess the impact of the metaverse-based family sculpture and meditation techniques on anxiety levels. The internal consistency reliability (Cronbach’s alpha) was 0.91 for the pre-test and 0.83 for the post-test.

#### 2.2.3 Patient Health Questionnaire-9

The Patient Health Questionnaire-9 (PHQ-9), developed by Kroenke et al. (2001), is a nine-item self-report inventory of the frequency of depressive symptoms experienced over the past 2 weeks. Each item is rated on a 4-point Likert scale (0 = not at all; 3 = nearly every day), with total scores ranging from 0 to 27. Higher scores indicate greater severity of depression. This study used the PHQ-9 to evaluate changes in depressive symptoms resulting from the metaverse-based techniques. The internal consistency reliability (Cronbach’s alpha) was 0.85 for the pre-test and 0.82 for the post-test.

#### 2.2.4 Program satisfaction

Program satisfaction was assessed through structured post-intervention interviews. Participants provided subjective feedback on program satisfaction, perceived effectiveness, suggestions for improvement, and overall impression (Table 2). Satisfaction was quantified using five items rated on a 7-point Likert scale (1 = not at all; 7 = very much).

**Table 2:** Program satisfaction scale.

Item	Description
1	Do you think this program was helpful for emotional stability and self-understanding?
2	Compared with baseline, by how much do you feel your emotional stability has improved?
3	To what extent do you think the metaverse is effective as a psychological care tool?
4	Would you recommend this program to your acquaintances?
5	Would you be willing to participate in a similar program in the future?

Using the PSS, GAD-7, and PHQ-9, participants’ stress, depression, and anxiety levels were evaluated during both pre- and post-intervention programs. After completing the program in each intervention group, satisfaction was assessed to provide a comprehensive analysis of the effectiveness of each program.

### 2.3 Data analysis

The collected data were analyzed using the R program, applying a range of statistical techniques, including descriptive statistics and pre-post comparison analysis.

Descriptive statistics revealed baseline characteristics like age, gender, and family composition. Stress (PSS), anxiety (GAD-7), and depression (PHQ-9) levels were also measured to ensure group homogeneity. A paired t-test compared pre- and post-intervention scores, evaluating the impact of metaverse-based interventions on stress reduction. Effect sizes were calculated to determine the findings’ practical significance.

### 2.4 Program implementation

To ensure a fully controlled therapeutic experience, the interventions were conducted in a desktop-based 3D virtual environment called Meta-Counseling, developed by Etribe (Seoul, South Korea). While the platform incorporates elements commonly associated with metaverse technologies—including persistent virtual worlds, avatar-based interaction, and real-time social presence—participants accessed the environment through conventional desktop computers rather than immersive VR/AR headsets. The platform required minimum technical specifications (Intel i5 processor, 8GB RAM, Windows 10 or later) and utilized standard mouse and keyboard interfaces.

This setup enabled controlled experimental conditions and standardized protocols across participants, allowing them to manipulate objects, position family representations, and engage in guided activities while maintaining communication with therapists. While the desktop-based interface differs from head-mounted display systems in terms of immersive modality, it was deliberately chosen to ensure broad accessibility and minimize technical barriers while maintaining the core spatial and interactive features essential for family sculpture and meditation exercises.

**Figure 1:**
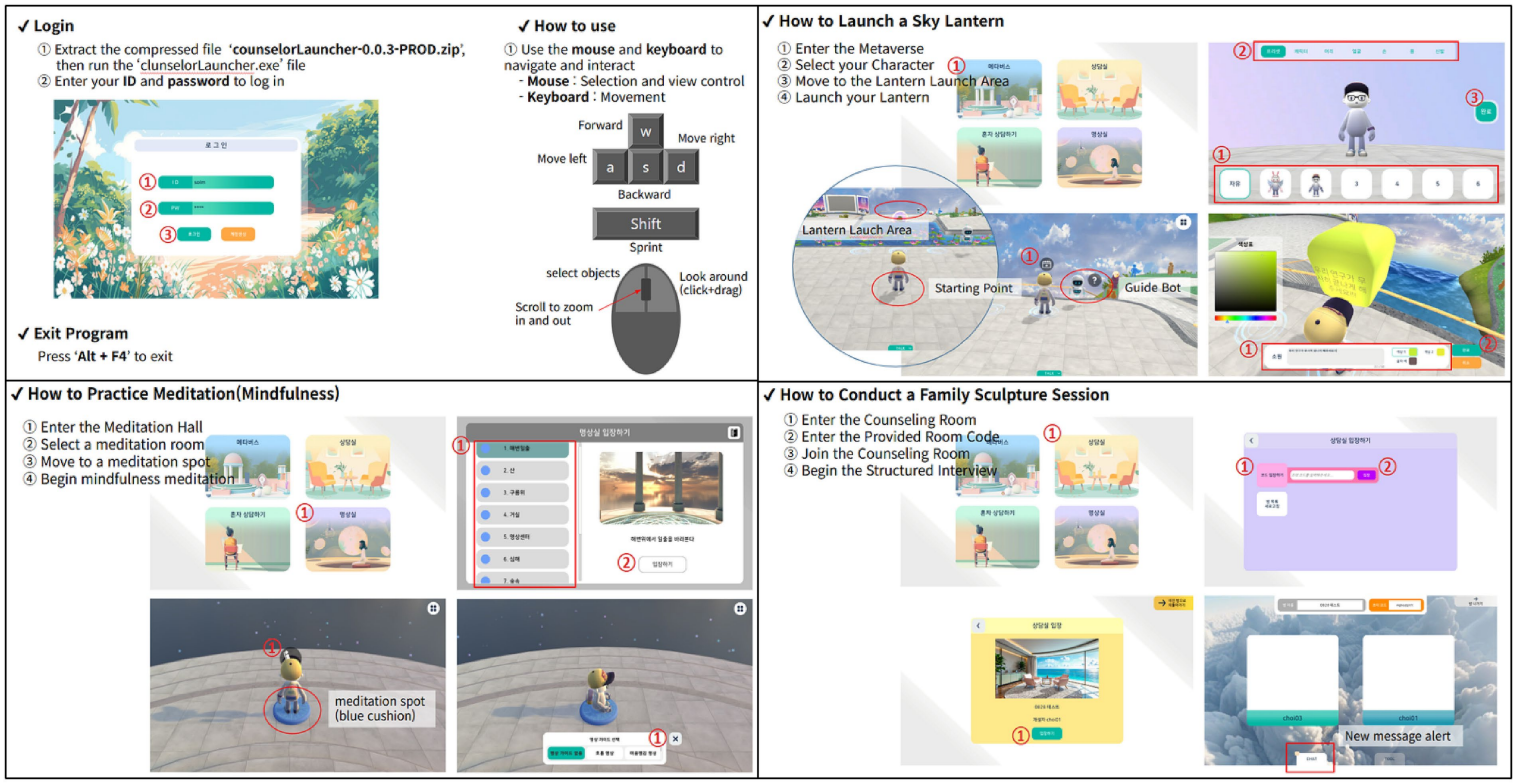
Step-by-step guide to using Meta-Counseling. This figure presents the installation and operation guide provided during the orientation session for study participants. The original image includes Korean text, as it was designed for native Korean speakers.

Prior to the study, participants installed the Meta-Counseling program and received technical onboarding to become familiar with the platform interface, session procedures, and mouse-based controls. A technical guide was provided (Figure 1), which included instructions on login procedures, interface navigation, and task execution. In addition, each participant’s understanding of the system was confirmed, and real-time technical support was provided throughout the study via chat or screen sharing to address any usability issues.

**Figure 2:**
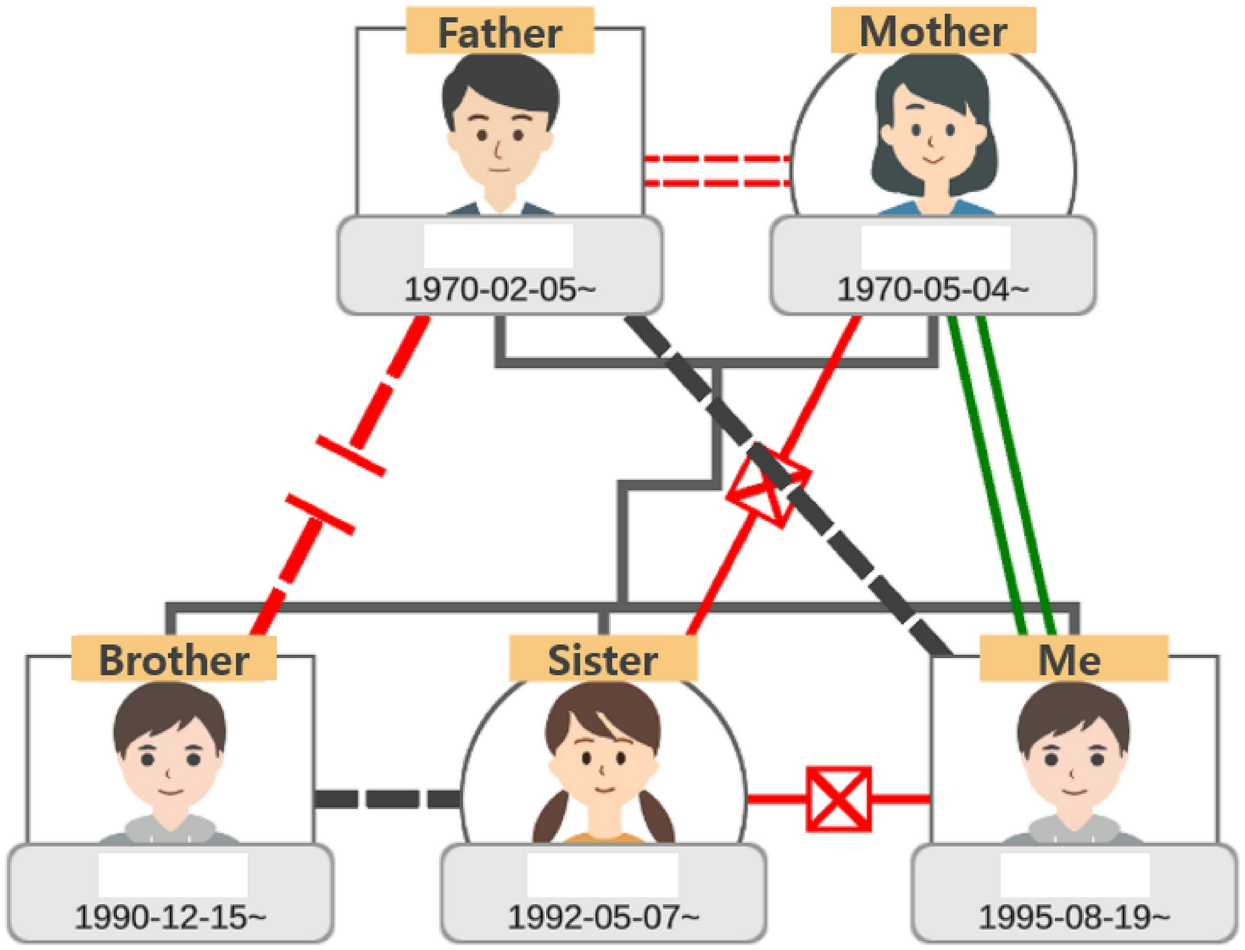
Genogram exploration. This scene depicts the genogram exploration conducted during the first intervention session.

**Figure 3:**
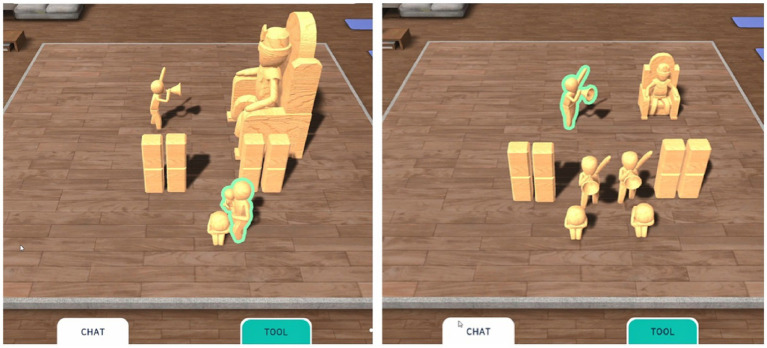
Arrangement and modification of the family sculpture. Screenshot from weeks 2 and 3 of the family sculpture sessions, showing the process of placing and adjusting virtual representations of family members.

**Figure 4:**
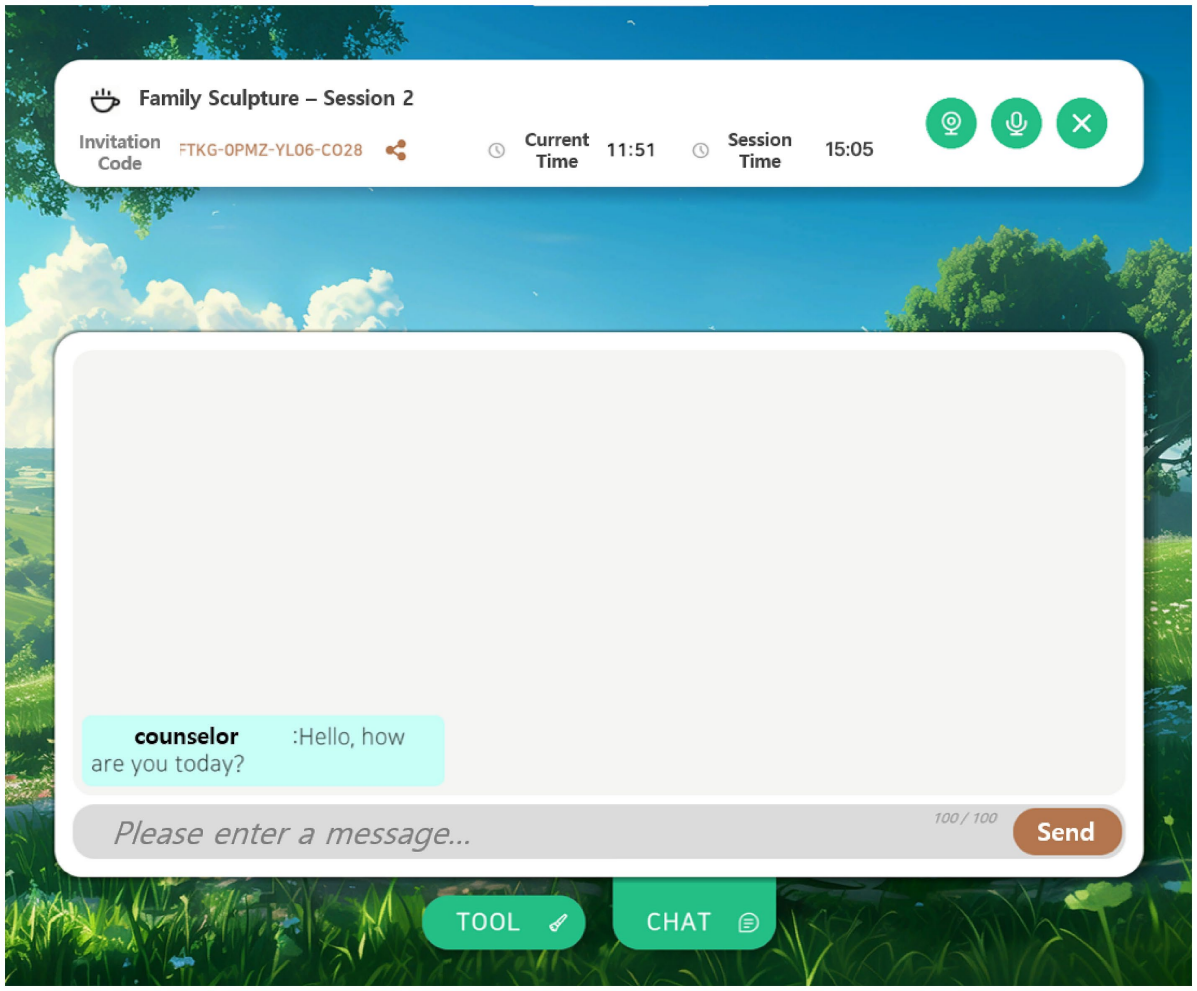
Counselor–client chat interaction. Chat exchanges that took place during the family sculpture sessions. The counselor facilitated structured therapeutic activities through the chat interface.

The groups engaged in different activities:

Family Sculpture Group: participants had weekly 15-min structured sessions over four weeks, facilitated by professional counselors through live chat in the platform. The sessions followed a thematic sequence (Table 3): (1) exploring the genogram (Figure 2), (2) constructing a visual family structure using mouse-based placement of figures in a 3D space, (3) modifying the arrangement to reflect perceived relational changes (Figure 3), and (4) insight and summarization through counselor prompts (Figure 4).

**Table 3:** Step-by-step process of the metaverse-based family sculpture intervention.

Step	Session focus	Participant activity	Facilitator role
1	Genogram Exploration	Identify and name key family members using an on-screen genogram tool	Provide initial guidance and clarification via chat
2	Family Sculpture Construction	Arrange family figures in the 3D space using the drag-and-drop interaction with a mouse to reflect relationships	Monitor activity and offer prompts via chat
3	Modification and Re-structuring	Adjust placements to represent desired relational changes or emerging insights	Ask reflective questions to deepen understanding
4	Reflection and Summary	Reflect on the overall structure and emotional meaning of the arrangement	Facilitate emotional processing and summarization

All sessions were conducted in a virtual counseling room using structured chat guidance and mouse-based interaction. No avatars were used.

**Figure 5:**
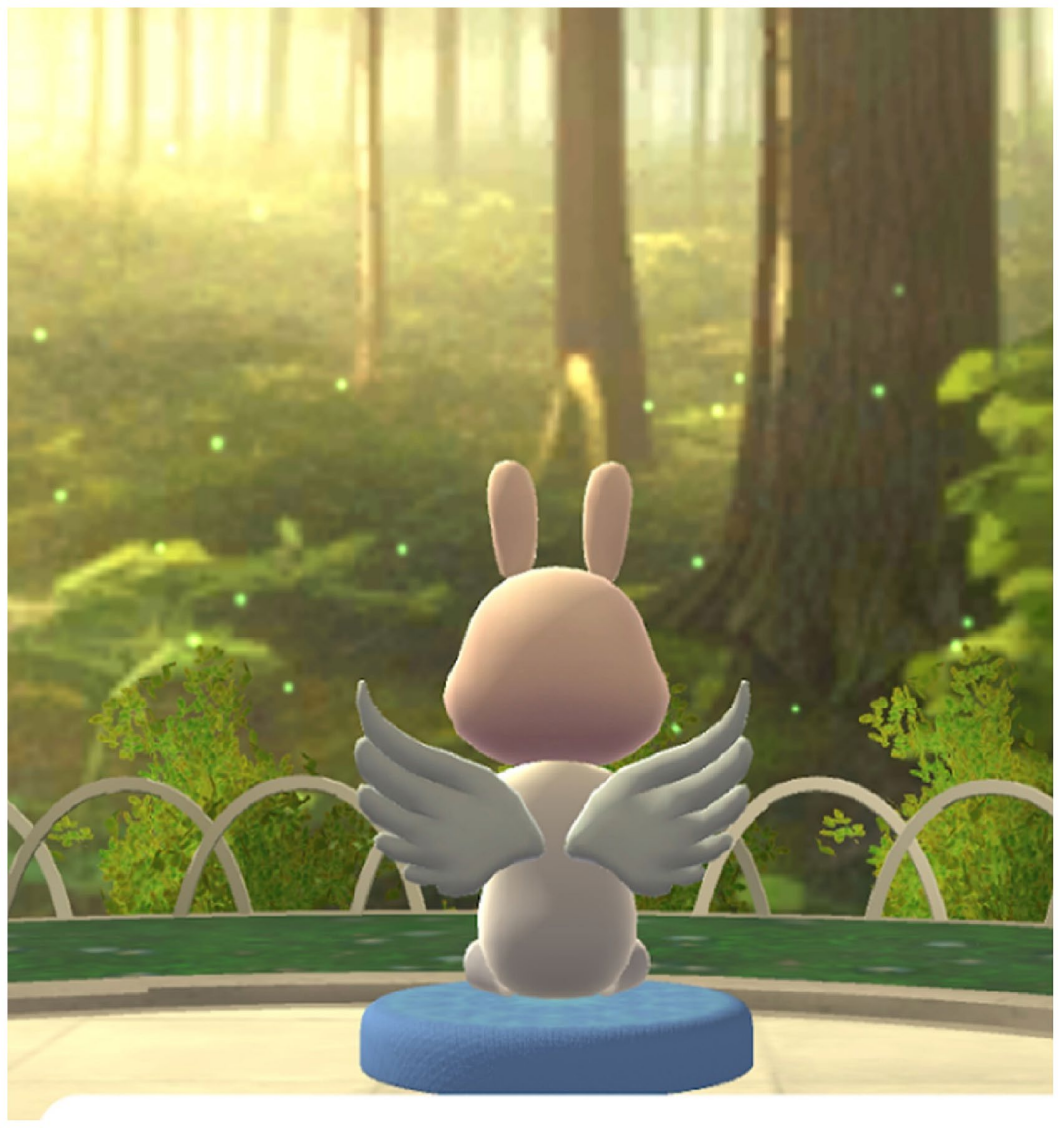
Participant engaging in meditation within the metaverse environment. This figure shows a participant using an avatar to engage in guided meditation within the metaverse platform. The immersive environment was designed to support emotional regulation and attentional focus through mindfulness practice.

**Figure 6:**
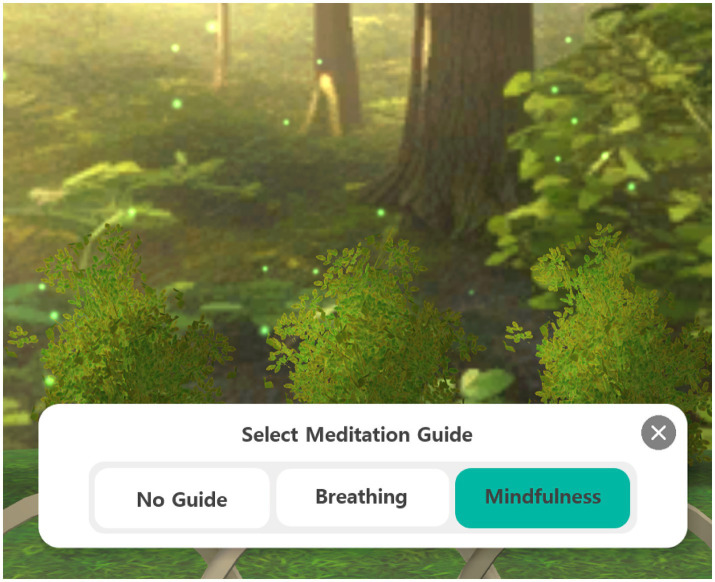
Delivery of mindfulness meditation. Participants accessed a virtual meditation room and received guided mindfulness meditation via the in-app menu interface.

Meditation Group: participants participated in guided meditative sessions for 5 mins per session, 2–3 times per week (Figure 5). The virtual environment featured calming visual and auditory stimuli (e.g., forest, temple, ocean), and participants were encouraged to use earphones for enhanced immersion. Mindfulness breathing techniques were delivered through embedded audio guidance (Figure 6).

Combined meditation and family sculpture (M + FS) Group: participants engaged in both meditation sessions and the weekly family sculpture activities as described above.

No-Intervention Group: participants were instructed not to engage in any form of psychological counseling, meditation, relaxation training, or self-care activities during the study period. This guidance was communicated during onboarding and through written materials, and adherence was monitored through weekly check-ins.

**Figure 7:**
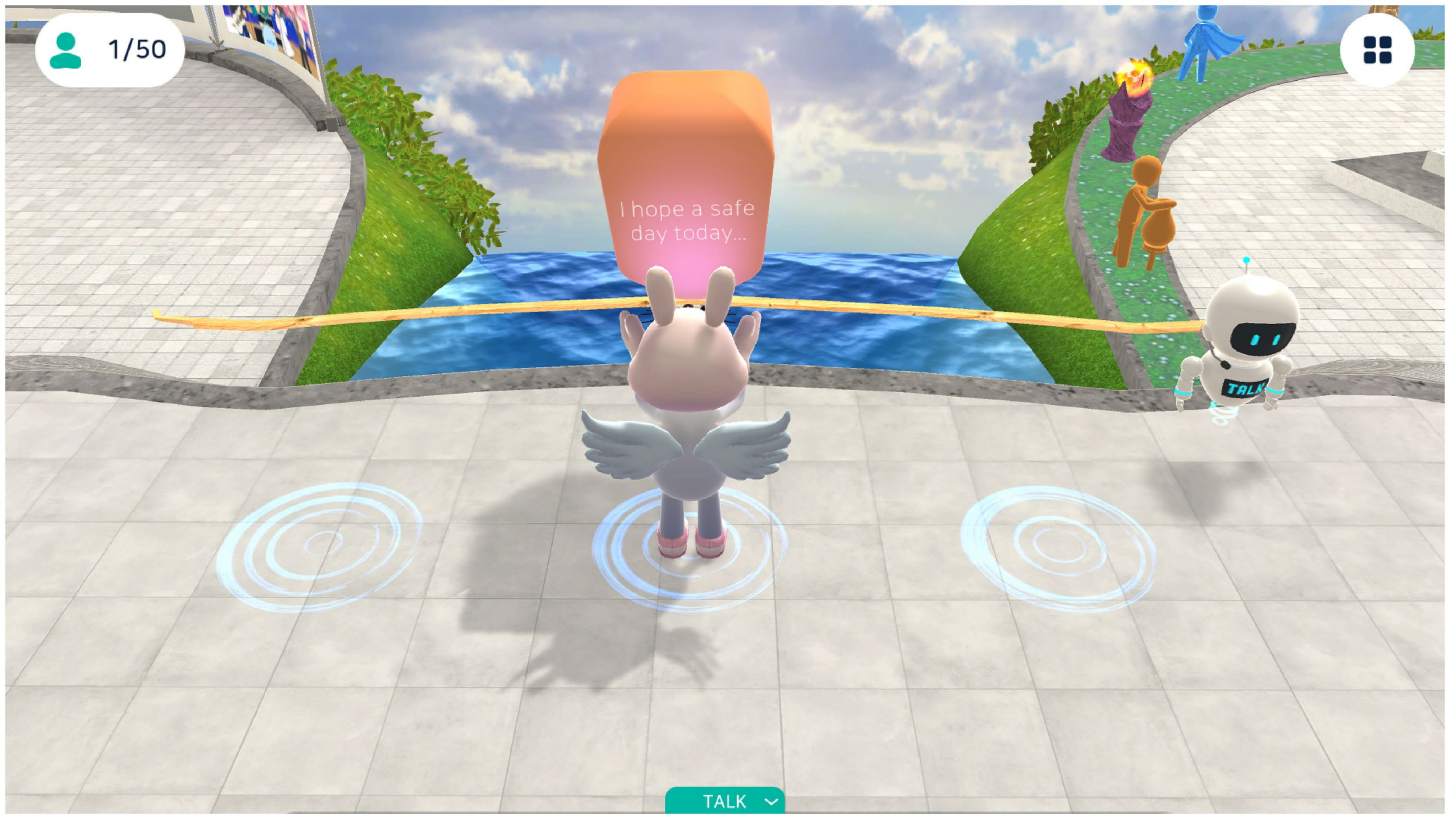
Wish lantern activity in the metaverse. In this scene, participants used their avatars to release a virtual lantern containing written wishes. Designed as a placebo intervention, this activity aimed to control for nonspecific effects such as participation and expectancy, while promoting emotional release and symbolic expression in an immersive setting.

Placebo Group: participants took part in a wish lantern activity within the metaverse, releasing virtual lanterns containing written wishes 2–3 times per week (Figure 7). This activity, designed as a placebo, involved minimal emotional engagement to simulate participation.

All participants completed pre- and post-intervention assessments using validated tools to evaluate changes in anxiety and program satisfaction. The study adhered to strict ethical guidelines ensuring anonymity, confidentiality, and voluntary participation.

Although participants were all adult office workers with sufficient computer literacy, a few initially reported minor difficulties in navigating the metaverse environment. To address this, real-time technical support was provided throughout the study. As the intervention progressed, repetitive tasks helped minimize usability differences across participants. Therefore, differences in digital fluency were not considered to have significantly influenced the outcomes.

## 3 Results

### 3.1 Descriptive statistics

Before examining the differences between groups, descriptive statistics for pre- and post-tests of each group were analyzed (Table 4).

**Table 4:** Results of descriptive statistical analysis.

Group		*n*	Pre			Post		
			Total	Mean	SD	Total	Mean	SD
No intervention	PSS	19	302	15.895	4.886	307	16.158	4.388
	GAD-7		48	2.526	1.389	50	2.632	1.802
	PHQ-9		58	3.053	1.649	63	3.316	2.358
Wish lantern	PSS	14	216	15.429	4.519	234	16.714	6.256
	GAD-7		37	2.643	1.646	41	2.929	1.817
	PHQ-9		49	3.500	2.534	43	3.071	2.464
Meditation	PSS	15	315	21.000	5.028	274	18.267	5.898
	GAD-7		97	6.467	4.984	64	4.267	3.634
	PHQ-9		94	6.267	5.244	66	4.400	3.501
Family sculpture	PSS	18	342	19.000	5.891	319	17.722	6.027
	GAD-7		86	4.778	4.011	68	3.778	3.993
	PHQ-9		97	5.389	4.937	91	5.056	5.286
M + FS	PSS	13	241	18.538	5.395	202	15.535	7.457
	GAD-7		62	4.563	6.930	28	2.875	2.544
	PHQ-9		61	5.063	5.105	36	3.313	3.004

### 3.2 Normality test

To conduct a paired t-test, normality assumption for the pre-post mean difference in each group must be fulfilled. The results of the Shapiro–Wilk test (Table 5) showed that normality assumption was violated for most variables (*p* < 0.05). Consequently, the Wilcoxon signed-rank test, a nonparametric alternative, was used for all within-group comparisons. This method does not assume normal distribution and is therefore more appropriate for small sample sizes, providing robust and reliable statistical estimates. The results of these analyses for each group are presented in Table 6.

**Table 5:** Shapiro–Wilk test result.

Group	*n*	PSS		GAD-7		PHQ-9	
		Estimate	*p* value	Estimate	*p* value	Estimate	*p* value
No intervention	19	0.939	0.249	0.889	0.031	0.929	0.169
Wish lantern	14	0.938	0.399	0.944	0.470	0.902	0.120
Meditation	15	0.868	0.032	0.856	0.021	0.856	0.021
Family sculpture	18	0.945	0.353	0.906	0.073	0.919	0.124
M + FS	13	0.946	0.533	0.584	<0.001	0.751	0.002

**Table 6:** Pre-post changes by intervention group.

Group	Scale	Pre-test M (SD)	Post-test M (SD)	MD (95% CI)	Cohen’s d (95% CI)	*p*-value
No intervention	PSS	15.90 (4.2)	16.16 (4.8)	0.26 (−1.2, 1.7)	0.06 (−0.26, 0.38)	0.775
No intervention	GAD-7	2.53 (3.1)	2.63 (3.4)	0.11 (−0.8, 1.0)	0.03 (−0.25, 0.31)	0.631
No intervention	PHQ-9	3.05 (2.8)	3.32 (3.2)	0.26 (−0.6, 1.1)	0.09 (−0.21, 0.39)	0.654
Wish lantern	PSS	15.43 (5.1)	16.71 (4.9)	1.29 (−0.2, 2.8)	0.26 (−0.04, 0.56)	0.326
Wish lantern	GAD-7	2.64 (2.9)	2.93 (3.1)	0.29 (−0.5, 1.1)	0.10 (−0.17, 0.37)	0.650
Wish lantern	PHQ-9	3.50 (3.0)	3.07 (2.8)	−0.43 (−1.3, 0.4)	−0.15 (−0.43, 0.13)	0.189
Meditation	PSS	21.00 (6.2)	18.27 (5.8)	−2.73 (−5.1, −0.4)	**−0.46 (−0.85, −0.07)**	0.022*
Meditation	GAD-7	6.47 (4.1)	4.27 (3.5)	−2.20 (−4.1, −0.3)	**−0.58 (−1.09, −0.07)**	0.076
Meditation	PHQ-9	6.27 (3.8)	4.40 (3.2)	−1.87 (−3.6, −0.1)	**−0.52 (−1.01, −0.03)**	0.041*
Family sculpture	PSS	19.00 (5.4)	17.72 (4.9)	−1.28 (−2.8, 0.2)	−0.25 (−0.55, 0.05)	0.260
Family sculpture	GAD-7	4.78 (3.2)	3.78 (2.8)	−1.00 (−1.9, −0.1)	−0.33 (−0.63, −0.03)	0.015*
Family sculpture	PHQ-9	5.39 (3.4)	5.06 (3.1)	−0.33 (−1.2, 0.5)	−0.10 (−0.36, 0.16)	0.421
M + FS	PSS	18.54 (5.2)	15.54 (4.8)	−3.00 (−4.4, −1.6)	**−0.60 (−0.88, −0.32)**	0.049*
M + FS	GAD-7	4.77 (3.1)	2.15 (2.2)	−2.62 (−3.8, −1.4)	**−0.96 (−1.39, −0.53)**	0.021*
M + FS	PHQ-9	4.69 (2.9)	2.77 (2.1)	−1.92 (−2.9, −0.9)	**−0.74 (−1.12, −0.36)**	0.044*

Effect size is reported as Cohen’s d (small: 0.2, medium: 0.5, large: 0.8); 95% confidence intervals (CI) are provided for both mean differences (MD) and effect sizes; M = mean, SD = standard deviation.

Bold values indicate statistically significant results (*p* < 0.05) or effect sizes approaching or exceeding a medium threshold (approximately |d| ≥ 0.5).

### 3.3 Pre-post change analysis

The combined intervention group (M + FS) demonstrated the most consistent and significant improvement effects. Statistically significant reductions were observed across all indicators: stress (PSS: MD = −3.00, 95% confidence interval [CI] [−4.4, −1.6], *p* = 0.049, *d* = −0.60), anxiety (GAD-7: MD = −2.62, 95% CI [−3.8, −1.4], *p* = 0.021, *d* = −0.96), and depression (PHQ-9: MD = −1.92, 95% CI [−2.9, −0.9], *p* = 0.044, *d* = −0.74). Notably, the anxiety indicator showed a large effect size (*d* = −0.96), indicating clinically meaningful improvement.

The meditation-only group showed statistically significant reductions in stress (PSS: MD = −2.73, 95% CI [−5.1, −0.4], *p* = 0.022, *d* = −0.46) and depression (PHQ-9: MD = −1.87, 95% CI [−3.6, −0.1], *p* = 0.041, *d* = −0.52), while anxiety (GAD-7: MD = −2.20, 95% CI [−4.1, −0.3], *p* = 0.076, *d* = −0.58) approached statistical significance.

The family sculpting-only group showed statistically significant reduction only in anxiety (GAD-7: MD = −1.00, 95% CI [−1.9, −0.1], *p* = 0.015, *d* = −0.33), although the effect size was small (*d* = −0.33). No significant changes were observed in stress or depression. Neither the placebo group (wish lantern making) nor the no-intervention control group showed statistically significant changes in any psychological indicators.

### 3.4 Between-group difference testing

Kruskal-Wallis tests were conducted to examine between-group differences; overall between-group differences were not statistically significant. However, Mann–Whitney U tests comparing control and placebo groups with intervention groups revealed significant differences. Nevertheless, the differences between combined interventions and single interventions were not statistically significant (Table 7).

**Table 7:** Treatment effectiveness analysis.

Variable	Control/placebo vs. intervention			Single vs. combined treatment		
	Estimate	*p* value	FDR-adjusted *p*	Estimate	*p* value	FDR-adjusted *p*
Depression	972	0.032	0.048*	258.5	0.281	0.421
Anxiety	1,054	0.003	0.009**	219	0.921	0.921
Stress	1034.5	0.006	0.018*	247.5	0.424	0.636

Estimates represent Mann–Whitney U statistics; *FDR-adjusted *p* < 0.05, **FDR-adjusted *p* < 0.01.

To control for increased Type I error due to multiple comparisons, the Benjamini-Hochberg False Discovery Rate (FDR) correction was applied and results were interpreted based on the corrected q-values (*q* = 0.05). FDR was chosen over more conservative methods like the Bonferroni correction to balance Type I error control with statistical power, particularly given the exploratory nature of this intervention study (Benjamini and Hochberg, 1995).

### 3.5 Effect size analysis

Effect size analysis using Cohen’s d provides important indicators for evaluating the practical effects and clinical significance of interventions beyond statistical significance (Table 6). The M + FS group achieved medium to large effect sizes across all psychological indicators: stress (PSS: *d* = −0.60), anxiety (GAD-7: *d* = −0.96), and depression (PHQ-9: *d* = −0.74). The large effect size for anxiety (*d* = −0.96) indicates clinically meaningful improvement that would be perceptible in daily life according to Cohen’s (1988) criteria.

The meditation-only group achieved medium effect sizes in stress (PSS: *d* = −0.46) and depression (PHQ-9: *d* = −0.52), indicating substantial benefits for inner emotional regulation and stress relief. Anxiety (GAD-7: *d* = −0.58) also showed a medium effect size in the meditation-only group despite not reaching statistical significance.

The family sculpting-only group showed a small effect size in anxiety (GAD-7: *d* = −0.33) but very limited effects in stress (PSS: *d* = −0.25) and depression (PHQ-9: *d* = −0.10), suggesting effectiveness for relationship-based anxiety but limitations in overall emotional regulation.

Both placebo and control groups showed negligible effect sizes (*d* < 0.2) across all indicators, confirming no substantial change.

These results clearly demonstrate the superiority of the combined intervention and suggest that cases showing medium effect sizes, despite a lack of statistical significance, may yield significant results in future studies with larger samples or extended intervention periods.

### 3.6 Program satisfaction

Table 8 summarizes participants’ evaluations of program effectiveness, usability, and subjective satisfaction.

**Table 8:** Results of program satisfaction survey.

Item	Total	Wish lantern	Meditation	Family sculpture	M + FS
1	5.05	4.93	5.47	4.39	5.50
2	4.57	4.57	5.00	3.78	5.06
3	4.90	5.07	4.87	4.50	5.25
4	4.83	4.86	5.07	4.11	5.38
5	5.59	5.57	5.73	5.11	6.00

The survey consists of a 7-point Likert scale with the following items: 1. Degree of perceived help with emotional stabilization and self-understanding; 2. Level of emotional stabilization before and after program participation; 3. Usability of the metaverse for psychological care content in the program; 4. Likelihood to recommend the program based on satisfaction; 5. Interest in participating in the program again.

Across the groups, the family sculpture group reported the lowest mean satisfaction score, while the combined meditation and family sculpture (M + FS) group received the highest satisfaction ratings.

## 4 Discussion

This study analyzed metaverse-based psychological interventions—family sculpture and meditation, both individually and in combination—to evaluate their impact on stress (PSS), anxiety (GAD-7), and depression (PHQ-9). Significant differences were observed in psychological outcomes depending on the intervention type, with the combined meditation and family sculpture group (M + FS) demonstrating the highest overall effectiveness. The key findings are summarized below.

First, the meditation group showed significant reductions in stress (PSS) and depression (PHQ-9) but did not show statistical significance for anxiety (GAD-7). These findings are consistent with previous research demonstrating that meditation is effective in alleviating psychological stress and improving emotional regulation (Shapiro et al., 2004; Gan et al., 2022). Similarly, Bhardwaj et al. (2023) reported that a 12-week mobile-based meditation and breathing program reduced burnout and improved job satisfaction among healthcare professionals, which aligns with the stress-reduction effects observed in the present study. However, the absence of immediate anxiety reduction suggests that anxiety improvement through meditation may require long-term practice and accumulated experience. According to mindfulness theory, attentional control and present-moment awareness develop gradually through sustained practice, and anxiety reduction may require more time and repeated experience than stress or depression reduction (Bishop et al., 2004). This temporal dimension of therapeutic change will be an important consideration for future program design and expectation setting.

Second, the family sculpting group showed n reduction only in anxiety (GAD-7), with no notable changes observed in stress and depression. This suggests that visualizing and reconstructing family relationships is effective in addressing fundamental emotional anxiety (Satir, 1998; de Bernart and Mariotti, 2021). This selective effect aligns with Satir’s (1998) family systems theory and provides empirical support for family sculpting as an effective technique for addressing relational dynamics and relationship-based anxiety. These findings are consistent with Jefferson’s (1978) early observations that while family sculpting is a powerful experiential therapeutic tool, it may have limitations in individual emotion regulation. de Bernart and Mariotti (2021) emphasized that visual representation elicits unconscious family dynamics that are difficult to access through verbal expression; the present study demonstrates that this visualization mechanism operates effectively even in metaverse environments.

Third, the M + FS group demonstrated statistically significant reductions across all psychological indicators, with greater magnitude of reduction compared to individual intervention groups. This demonstrates that meditation and family sculpting produce complementary and synergistic effects, with meditation enhancing inner tranquility and balance while family sculpting addresses anxiety within relational contexts. These results align with Cuijpers et al.’s (2013) meta-analysis of multicomponent psychotherapy and represent the first case of extending the existing theory that utilizing multicomponent approaches in metaverse-based interventions leads to more robust therapeutic outcomes compared to using single techniques. According to von Sydow et al.’s (2024) systematic psychotherapy framework, therapeutic effects are maximized through the interaction between intrapersonal and relational changes. The present study shows that meditation’s inner emotional regulation and family sculpting’s approach to relational anxiety function as complementary mechanisms.

Fourth, the effectiveness of metaverse-based interventions confirmed in this study aligns with Carl et al.’s (2019) meta-analysis of VR exposure therapy, which reported that VR therapy could demonstrate equivalent or, in some cases, superior therapeutic effects compared to traditional face-to-face therapy. The present study extends this evidence to mindfulness-based and systemic family intervention techniques, suggesting that VR-based interventions can serve as a viable platform for more integrated and multicomponent psychotherapy beyond simple behavioral exposure therapy. Riva et al.’s (2021) VR for inner and outer theory is useful in explaining the present study’s findings that meditation supports inner regulation, while family sculpting supports relational externalization, creating synergistic effects. The effective operation of creative mechanisms is consistent with findings reported by Kreijen and Abbing (2024) and Brandon and Goldberg (2017), who clarified that three-dimensional representation facilitates emotional processing and integration.

Fifth, the M + FS group showed the highest levels of satisfaction (emotional stabilization 4.57 points, metaverse technology ease of use 4.90 points) and future intervention participation willingness. This suggests, like Cho et al.’s (2024) research, that metaverse-based counseling can enhance accessibility and participation motivation while maintaining therapeutic effectiveness. In contrast, the relatively lower satisfaction in the family sculpting-only group can be attributed to the significant time requirements, structural complexity, and demands for emotional immersion. Family sculpting is not merely a visual activity but a high-level task that spatially reproduces and interprets family dynamics and emotional structures, which may have required higher cognitive and emotional concentration in the metaverse environment. The high satisfaction in the M + FS group appears to stem from participants’ ability to autonomously secure time for self-insight and emotional stabilization through meditation, while engaging in emotional experiences under expert guidance. This demonstrates how external interpretation (family relationship visualization) and internal awareness (mindfulness meditation) work complementarily to help participants achieve safer and more integrated psychological experiences.

Lastly, participants in this study reported moderate to above-moderate levels of stress and mild levels of emotional difficulties, which can be interpreted as a general adult population with psychological vulnerabilities rather than a typical clinical population. The absence of significant changes in the no-treatment control group indicates that psychological improvement is unlikely to occur spontaneously without targeted intervention.

These findings suggest that even everyday psychological difficulties require structured interventions focused on specific domains such as emotion regulation or relationship issues. In particular, the results point to the possibility of personalized interventions tailored to individual psychological characteristics and symptom types, namely precision psychotherapy.

This study holds academic significance as it broadens the scope of traditional psychotherapy techniques by incorporating the cutting-edge metaverse technology. Notably, it demonstrates the effectiveness of combining meditation and family sculpture techniques, providing robust evidence that a multicomponent approach is effective in addressing psychological issues. The findings herein highlight the potential of metaverse-based psychological interventions in contributing to the development of psychological care content and clinical applications.

### 4.1 Limitations

While this study provides promising evidence for the effectiveness of a metaverse-based psychological intervention combining family sculpture and meditation, some limitations must be acknowledged.

First, the study sample consisted of self-selected participants with relatively high digital literacy and familiarity with virtual environments. This introduces a potential selection bias and limits the generalizability of the findings to broader populations, particularly those with limited access to or comfort with immersive technology. Future research should aim to recruit more diverse samples with varying levels of technological proficiency.

Second, the intervention period was relatively short (4 weeks), which may have constrained the full therapeutic potential of the intervention. Psychological change, particularly in systemic or mindfulness-based approaches, often requires sustained engagement over time. In addition, the absence of follow-up assessments precludes conclusions about the long-term maintenance of treatment effects.

Third, although technical support was provided throughout the sessions, differences in participants’ ability to engage with the digital interface may have influenced emotional expressiveness and intervention adherence. As the use of immersive platforms grows in mental health care, individual variability in digital engagement should be considered a potential moderating factor.

Fourth, the placebo condition using the wish lantern activity may not have been entirely psychologically inert. Symbolic acts such as articulating and releasing a wish could elicit unintended emotional effects, potentially weakening the contrast between active and control groups. Future studies should refine placebo activities to minimize residual therapeutic influence.

Fifth, all outcome measures relied on self-report instruments, which may be susceptible to expectancy effects or social desirability bias. To increase objectivity and validity, future research should consider incorporating physiological or observer-based assessments alongside self-report measures.

Although the intervention was conducted in a virtual environment described as “metaverse-based,” it did not involve the use of fully immersive hardware such as VR or AR headsets. Instead, participants accessed the platform through standard desktop or laptop computers, which inherently limits the depth of sensory engagement and presence compared to head-mounted displays. This discrepancy in immersiveness may have influenced participants’ emotional involvement and the overall therapeutic impact. Therefore, caution is warranted when generalizing the findings to other metaverse platforms with higher levels of immersion. Future studies should directly compare different levels of virtual immersion to better understand how interface modality affects psychological outcomes.

Despite these limitations, the present study contributes to the emerging field of metaverse-based psychotherapy and highlights the potential of integrating immersive technologies into structured, multicomponent psychological interventions. Future investigations should employ longer intervention periods, follow-up assessments, and diverse participant groups to enhance internal and external validity.

## Data Availability

The datasets presented in this article are not readily available because the dataset includes sensitive psychological assessment data and qualitative transcripts from therapy sessions, which are not publicly shared in order to protect participant confidentiality, in compliance with IRB protocol (P01-202504-01-006). Requests to access the datasets should be directed to Sang-Hyun Yoo via email at yoo.sh@dankook.ac.kr.
